# A Risk Prediction Model Based on Lymph-Node Metastasis in Poorly Differentiated–Type Intramucosal Gastric Cancer

**DOI:** 10.1371/journal.pone.0156207

**Published:** 2016-05-26

**Authors:** Jeung Hui Pyo, Hyuk Lee, Byung-Hoon Min, Jun Haeng Lee, Min Gew Choi, Jun Ho Lee, Tae Sung Sohn, Jae Moon Bae, Kyoung-Mee Kim, Hyeon Seon Ahn, Sin-Ho Jung, Sung Kim, Jae J. Kim

**Affiliations:** 1 Department of Medicine, Samsung Medical Center, Sungkyunkwan University School of Medicine, Seoul, Korea; 2 Department of Surgery, Samsung Medical Center, Sungkyunkwan University School of Medicine, Seoul, Korea; 3 Department of Pathology, Samsung Medical Center, Sungkyunkwan University School of Medicine, Seoul, Korea; 4 Biostatistics and Clinical Epidemiology Center, Samsung Medical Center, Sungkyunkwan University School of Medicine, Seoul, Korea; 5 Department of Biostatistics and Bioinformatics, Duke University Medical Center, Durham, North Carolina, United States of America; The University of Hong Kong, CHINA

## Abstract

**Background and Aim:**

Endoscopic submucosal dissection (ESD) for undifferentiated type early gastric cancer is regarded as an investigational treatment. Few studies have tried to identify the risk factors that predict lymph-node metastasis (LNM) in intramucosal poorly differentiated adenocarcinomas (PDC). This study was designed to develop a risk scoring system (RSS) for predicting LNM in intramucosal PDC.

**Methods:**

From January 2002 to July 2015, patients diagnosed with mucosa-confined PDC, among those who underwent curative gastrectomy with lymph node dissection were reviewed. A risk model based on independent predicting factors of LNM was developed, and its performance was internally validated using a split sample approach.

**Results:**

Overall, LNM was observed in 5.2% (61) of 1169 patients. Four risk factors [Female sex, tumor size ≥ 3.2 cm, muscularis mucosa (M3) invasion, and lymphatic-vascular involvement] were significantly associated with LNM, which were incorporated into the RSS. The area under the receiver operating characteristic curve for predicting LNM after internal validation was 0.69 [95% confidence interval (CI), 0.59–0.79]. A total score of 2 points corresponded to the optimal RSS threshold with a discrimination of 0.75 (95% CI 0.69–0.81). The LNM rates were 1.6% for low risk (<2 points) and 8.9% for high-risk (≥2 points) patients, with a negative predictive value of 98.6% (95% CI 0.98–1.00).

**Conclusions:**

A RSS could be useful in clinical practice to determine which patients with intramucosal PDC have low risk of LNM.

## Introduction

Endoscopic resection for early gastric cancer (EGC) is one of the most advanced and representative techniques in the field of therapeutic endoscopy and is increasingly used worldwide [1,2]. Ruling out lymph node metastasis (LNM) (and the risk thereof) is a critical step prior to attempting endoscopic mucosal resection (EMR) or endoscopic submucosal dissection (ESD) [3]. Therefore, the application of this procedure is limited to differentiated-type EGC because of the higher risk of LNM associated with undifferentiated EGCs [4–6]. Gastrectomy with lymph node dissection is considered the treatment of choice in patients with undifferentiated EGCs [7].

Recently, ESD has been indicated for undifferentiated EGC with negligible risk of LNM based on the surgical resection findings of such EGCs [8–13]. Gotoda et al. [8,14] and Kunisaki et al. [15] both reported that intramucosal undifferentiated type EGCs without ulceration or lymphovascular invasion, and with diameters <2cm, had no LNM. However, controversy exists regarding the role of ESD in undifferentiated EGC [16,17], and there are no individual guidelines concerning this matter to date. We previously developed an LNM risk-prediction model for signet ring cell-type intramucosal gastric cancer [18]. Even among undifferentiated-type EGCs, poorly differentiated EGC has clinicopathological features that are less amenable to endoscopic treatment than are those of signet ring cell EGC [13]. Therefore, these two types of EGCs should be managed separately when planning endoscopic treatment, and not as a single type of tumor with undifferentiated histology.

To identify the risk factors predictive of LNM in undifferentiated EGCs, several studies have reported the results in signet ring cell-type EGC [19–22]. However, few studies have been conducted in early poorly differentiated adenocarcinoma (PDC), including submucosal cancer, which is associated with a definite LNM rate [7,13,15,23,24]. The aim of this study was to evaluate the clinicopathological factors predictive of LNM in intramucosal PDC and develop a risk scoring system (RSS) model for predicting LNM.

## Methods

### Study Population

We prospectively analyzed the collected data of patients who had been diagnosed with PDC and underwent curative gastrectomies with lymph node dissection at the Samsung Medical Center from January 2002 to July 2015. After excluding tumors with mixed histology, 2250 patients were confirmed as having pure poor differentiated-type T1 (tumor invasion confined to mucosa or submucosa) gastric cancers, and after additionally excluding patients with a history of surgery or endoscopic resection for gastric cancer (n = 35), and patients with multiple tumors (n = 30), 1,169 patients had T1a (mucosa-confined) PDC. The tumors were classified histologically according to the World Health Organization’s Classification of Tumors [25].

### Analysis of Clinical Outcomes

Surgical specimens were cut into 2-mm slices, and further cut into 4-μm slides for standard hematoxylin and eosin staining. The gross appearance of the tumor, tumor size, tumor depth, number of resected nodes, presence of lymphatic-vascular involvement (LVI), and the presence of LNM were assessed by an expert pathologist. The Japanese classification of gastric carcinoma was used to classify the gross type of tumors, and then were classified into four macroscopic types: elevated (I, IIa, I + IIa, IIa + IIb), flat (IIb), depressed (IIc, IIc + III, III), and mixed (others). The maximum diameter was recorded as the tumor size. The invasion depth was defined as M2 if the tumor was confined to the lamina propria, and M3 if the tumor infiltrated into the muscularis mucosa. The association between the various clinicopathological factors including sex, age, tumor location, gross appearance, tumor size, tumor depth, the presence of ulcer, the number of resected nodes, the presence of LVI, and the presence of LNM were examined. The tumors were staged according to the seventh edition of the American Joint Committee on Cancer Staging Manual (7th edition) [26]. This study was approved by the Institutional Review Board of Samsung Medical Center. Informed consent was not obtained because of the retrospective design. Patient records/information was anonymized and de-identified prior to analysis.

### Statistical Analysis

Logistic regression analysis was used to determine the independent predictors of LNM, and the performance was internally validated by using a split sample approach. The entire data was randomly partitioned into a training set and a test set of an equal size in order to have a near equal number of cases (LNM) and controls. Using the training set, we employed stepwise regression analysis with alpha = 5% as the insertion or deletion criterion to select the clinicopathological factors that correlated with LNM. To identify the optimal threshold of the tumor size, which was originally a continuous variable, we performed the receiver operating characteristic (ROC) curve analysis using the fitted model to find the clinically applicable cutoff value maximizing the sum of sensitivity and specificity. The scores were assigned by using the linear predictor of the logistic regression in the final model by dividing the beta coefficients with the smallest coefficients and rounding up to the nearest integer. The total score was the sum of the scores for each component. The fitted prediction model was validated by calculating the risk scores of the patients in the test set and the area under the curve (AUC) of the ROC that was generated by using the observed outcomes (LNM vs. no LNM) and predicted risk scores. The optimal cutoff point to partition the patients into high-risk and low-risk groups of the RSS in terms of clinical utility, sensitivity, specificity, accuracy, positive predictive value (PPV), and negative predictive value (NPV) was evaluated by using the ROC curve area and Youden index. Data were analyzed with SAS software version 9.4 (SAS Institute Inc., Cary, NC, USA) and R software version 3.1.2 (The R Foundation for Statistical Computing, Vienna, Austria).

## Results

### Characteristics of the Study Population

Of the 1,169 patients diagnosed with intramucosal PDC, 634 were men and 535 were women. Their mean age was 52.8 years (range 23–85). The rate of LNM was 5.2% (61/1,169). The clinicopathological factors and the presence of LNM were analyzed (Table 1). On univariate analysis, patient’s sex, tumor size, depth of invasion, and LVI was the significant factors associated with LNM. There were no significant difference in terms of age, tumor location, macroscopic type, and presence of ulcer. The mean number of lymph nodes examined was 40.4 [standard deviation (SD) = 13.7] for the LNM-negative group and 44.5 (SD = 14.2) for the LNM-positive group, a difference that was of borderline significance in the univariate analysis (P = 0.059), but was not significant in the multivariate analysis (P = 0.116). Among the 61 LNM positive patients, the distributions of N stage were as follow: 42 (68.9%) patients were N1 (1–2 positive nodes), 10 (16.4%) patients were N2 (3–6 positive nodes), and 9 (14.8%) patients were N3 (≥7 positive nodes). We determined the optimal clinically applicable tumor size cutoff value was 3.2cm by maximizing the sum of sensitivity and specificity (i.e. Youden’s index) from the training set. Therefore the tumors were stratified as ≥3.2 cm or <3.2 cm. Using tumor size alone, the area under the ROC curve for the training set was 0.674 (95% confidence interval [CI], 0.56–0.78). The cutoff value for the tumor size that showed no LNM was 1.0 cm (0/93).

**Table 1:** Characteristics of the Whole Population.

Factors		No LNM (n = 1108)	LNM (n = 61)	*P*-value*
		% (n)	% (n)	
Age (mean±SD)		52.9 ± 10.9	50.0 ± 10.8	0.482
Sex (male:female)		611: 497	23: 38	0.007
Tumor location				
	Upper	9.2 (102)	9.8 (6)	0.720
	Middle	44.3 (491)	44.3 (27)	0.560
	Lower	46.5 (515)	45.9 (28)	
Macroscopic type				
	Elevated	2.2 (24)	0.0 (0)	0.984
	Flat	19.2 (213)	13.1 (8)	0.766
	Depressed	52.8 (585)	54.1 (33)	
	Mixed	25.8 (286)	32.8 (20)	0.276
Ulcer				0.909
	No	6.1(1047)	93.8 (57)	
	Yes	93.9 (61)	6.2 (4)	
Tumor size		3.0 ± 2.0	4.2 ± 2.5	0.001
Depth of invasion				0.025
	Lamina propria	34.9 (387)	13.1 (8)	
	Muscularis mucosa	65.1 (721)	86.9 (53)	
Number of resected nodes (mean±SD)		40.4 ± 13.7	44.5 ± 14.2	0.059
LVI				0.006
	No	97.7 (1083)	80.3 (49)	
	Yes	2.3 (25)	19.7 (12)	

*By logistic regression analysis. LNM indicates lymph node metastasis; SD, standard deviation; EGC, early gastric cancer; EMR, endoscopic mucosal resection; ESD, endoscopic submucosal dissection; LVI, lymphatic-vascular involvement.

### Derivation of the Risk Model for Lymph Node Metastasis

The study population was randomly split into 584 patients in the training set and 585 patients in the test set. Patient characteristics in the two sets were well balanced, except for tumor size (S1 Table). The multivariate logistic regression analysis revealed that female sex (odds ratio [OR] = 3.99; 95% CI, 1.71–9.33; P = 0.001), a tumor size of ≥3.2 cm (OR = 3.18; 95% CI, 1.44–7.05; P = 0.004), muscularis mucosa (M3) invasion (OR = 3.08; 95% CI, 1.03–9.22; P = 0.045), and a positive LVI status (OR = 7.38; 95% CI, 2.30–23.74; P < 0.001) independently predicted LNM. The area under the ROC curve of the RSS was 0.69 (95% CI, 0.59–0.79) in the test set. Using a cutoff value of 0.035 for RSS (based on the Youden’s index from the training set) produced a sensitivity of 81%, specificity of 48%, PPV of 8%, and NPV of 98% in the test set. On the other hand, the areas under the ROC curves were 0.61 for tumor size, 0.54 for sex, 0.61 for depth of invasion, and 0.60 for LVI. The risk score was assigned by dividing the beta coefficient from the logistic regression model by 1.1 (Table 2). The presence of LVI was assigned 2 points, while female sex, a tumor size ≥ 3.2 cm, or muscularis mucosa invasion were equally assigned 1 point each. The final scores ranged from 0 to 5. LNM rate was assessed by combining patient sex, LVI, tumor size, and invasion depth (Table 3). The patients were divided into male and female groups, and then they were subdivided based on the presence or absence of LVI. In each group, the LNM rate was examined by the tumor size and the depth of invasion. In the male group, of the 189 patients with invasion limited to the lamina propria (M2), only 1 (0.5%) showed LNM. The rate of LNM increased markedly from 2.1% to 50.0% in the female group. The LNM rate also increased from 1.6% to 7.8% when the tumor size was ≥ 3.2 cm in male patients with muscularis mucosa (M3) invasion and those who did not have LVI, and 5.3% to 10.5% in the female group.

**Table 2:** Derivation of the Risk Scoring System for Lymph Node Metastasis Prediction.

Factors		OR (95% CI)	Beta	Standard error	Scores	*P*-value*
Sex						0.001
	Male	Reference				
	Female	3.99 (1.71–9.33)	1.3888	0.4336	1	
Tumor size						0.004
	< 3.2cm	Reference				
	≥ 3.2cm	3.18 (1.44–7.05)	1.1581	0.1054	1	
Depth of invasion						0.045
	Lamina propria	Reference				
	Muscularis mucosae	3.08 (1.03–9.22)	1.1248	0.5597	1	
LVI						<0.001
	No	Reference				
	Yes	7.38 (2.30–23.74)	1.9992	0.5960	2	

*By logistic regression analysis. All variables entered determined using three variables. OR indicates odd ratio; CI, confidence interval; LVI, lymphatic-vascular involvement.

**Table 3:** Lymph Node Metastasis Rate Assessed by Combining Sex, Lymphovascular Invasion, Tumor Size, and Depth of Invasion.

Tumor size (cm)			M2 (n, %)	M3 (n, %)
Male	LVI (-) (n = 614)	< 3.2	1/130 (0.8)	4/249 (1.6)
		≥ 3.2	0/56 (0)	14/179 (7.8)
	LVI (+) (n = 20)	< 3.2	0/3 (0)	1/6 (16.7)
		≥ 3.2	0/0 (0)	3/11 (27.3)
Female	LVI (-) (n = 518)	< 3.2	3/140 (2.1)	9/171 (5.3)
		≥ 3.2	3/64 (4.7)	15/143 (10.5)
	LVI (+) (n = 17)	< 3.2	1/2 (50.0)	5/9 (55.6)
		≥ 3.2	0/0 (0)	2/6 (33.3)

LVI indicates lymphatic-vascular involvement; M2, lamina propria; M3, muscularis mucosa.

### Optimal Threshold of the Risk Scoring System

**Fig 1:**
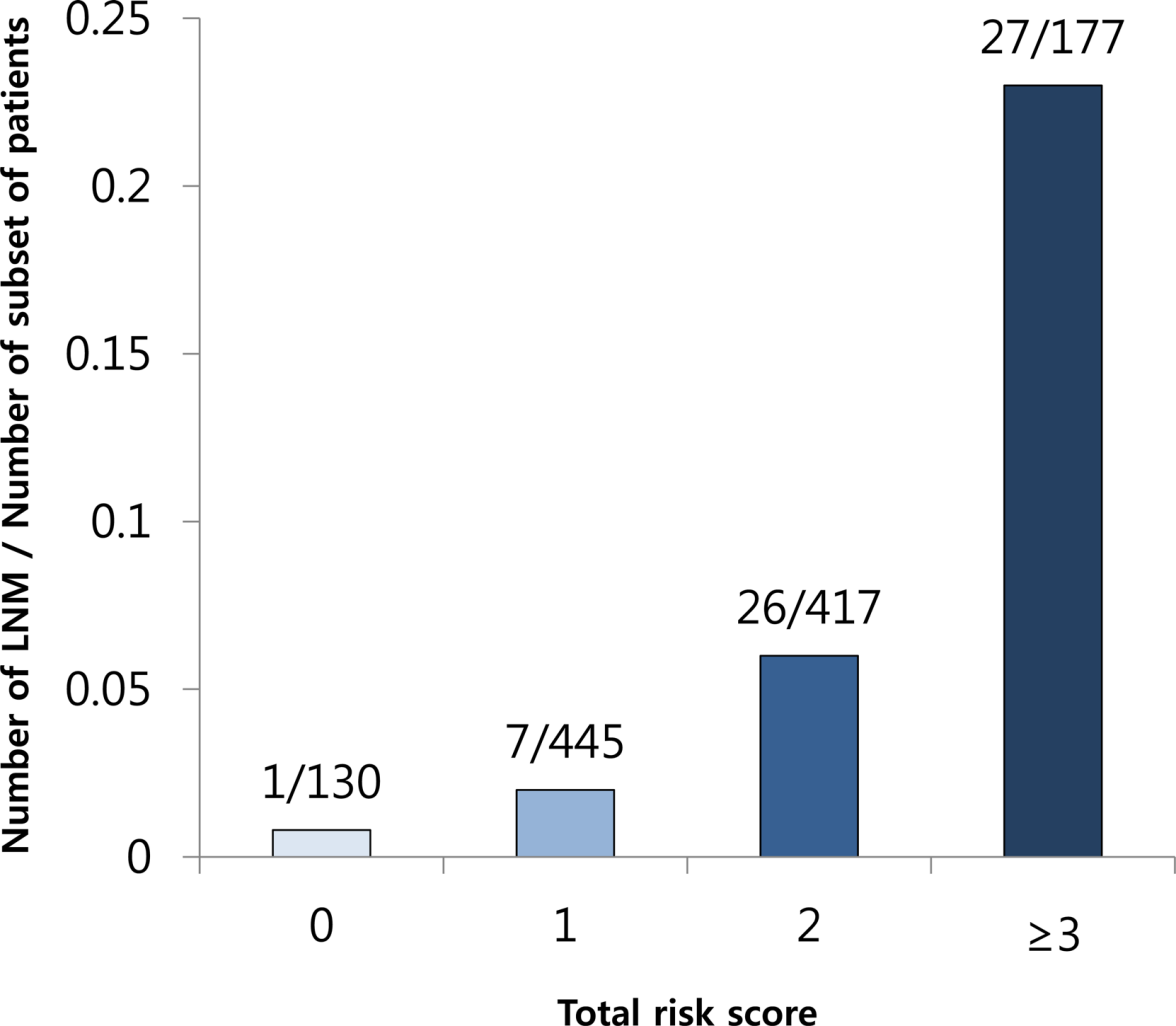
The number of lymph node metastases according to the total risk score. LNM, lymph node metastasis.

**Fig 2:**
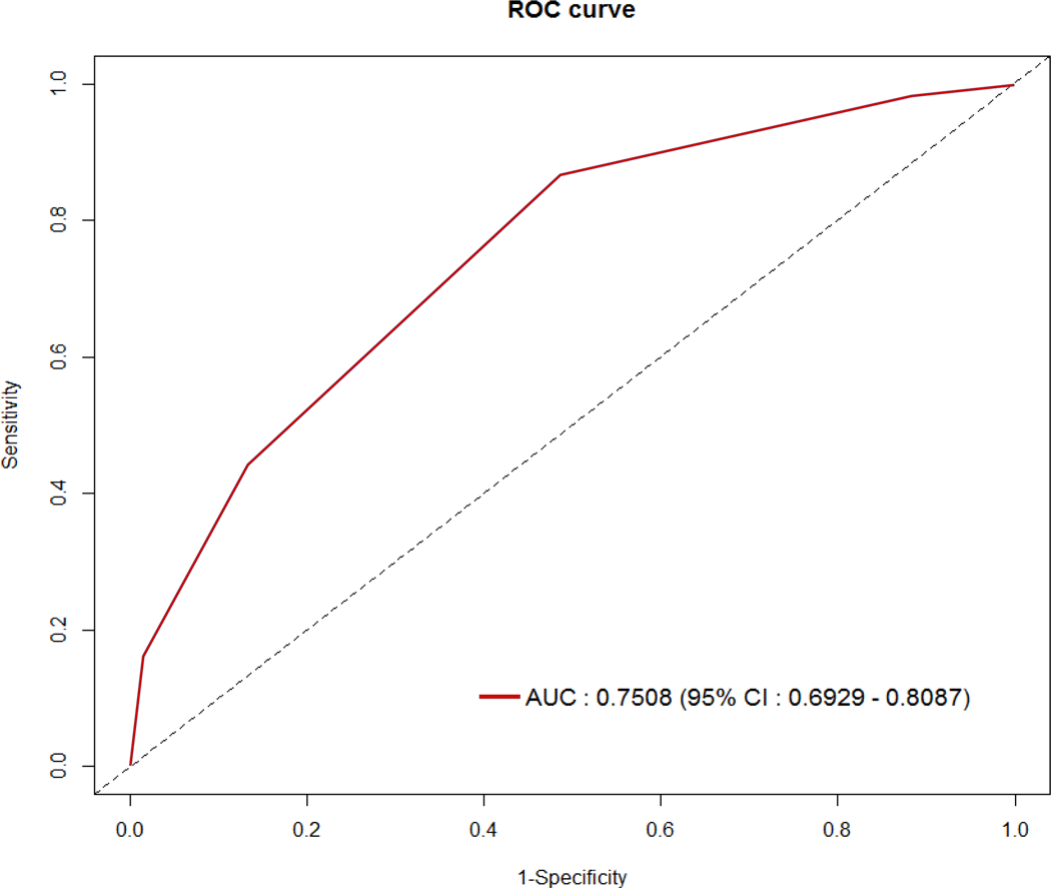
Receiver operating characteristic curve of the total score.

Given that our data provided a useful prediction model based on the training and validation sets, we fitted a refined prediction model by combining the training and test datasets. A total score of ≥2 points corresponded to the optimal threshold of the RSS in terms of clinical utility with an NPV of 98.6%. LNM were found in 1.4% (8/575) and 8.9% (53/594) in the low-risk and high-risk groups of the RSS, respectively (Fig 1). At this cutoff, the AUC of the ROC curve was 0.75 (95% CI 0.69–0.81) (Fig 2), and the sensitivity, specificity, PPV, NPV, and overall accuracy of the RSS were 89.9% (95% CI, 0.78–0.95), 51.2% (95% CI, 0.48–0.54), 8.9% (95% CI, 0.07–0.11), 98.6% (95% CI, 0.98–1.00), and 53.0% (95% CI, 0.50–0.56), respectively.

### Validation of the Risk Scoring System

**Fig 3:**
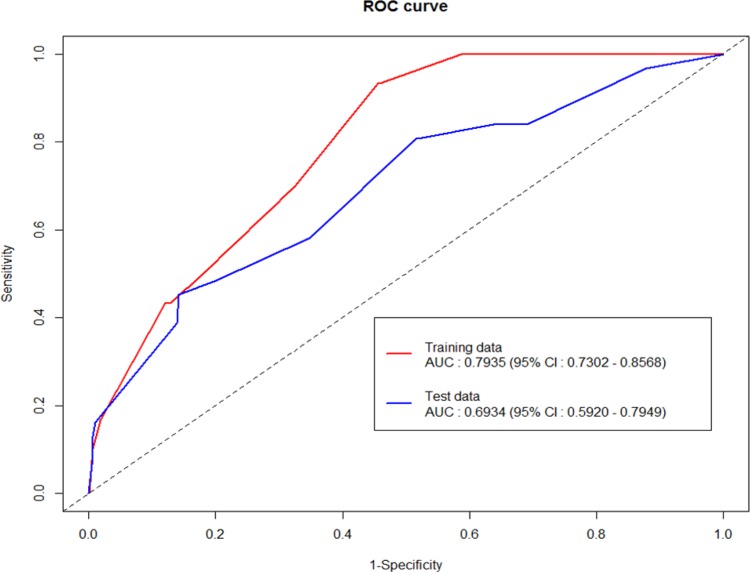
Receiver operating characteristic curve of the risk scoring system.

Internal validation was performed by applying the fitted model derived from the training set to the test set. The discriminatory power for this prediction model was moderate to good with an overall AUC of 0.69 (95% CI 0.59–0.79) in the ROC curve generated during internal validation (Fig 3). Therefore, the RSS that we developed was valid, and the performance of the RSS was analyzed by using all of the data.

## Discussion

In this study, we developed an RSS to predict LNM in mucosa-confined PDC. Although undifferentiated-type EGC is not generally accepted as a candidate for ESD, and PDC is considered less eligible for endoscopic treatment than is signet ring cell EGC [13], recent studies have suggested the cautious use of endoscopic treatment [7,11,15,23,27] or laparoscopic wedge resection [28] in selected cases with exceptionally low LNM. However, no specific guidelines have yet been developed for the use of ESD in cases of early PDC, and there is little evidence to support the oncological safety of ESD for early PDC.

The incidence of LNM in intramucosal (T1a) PDC has been reported to be between 2.2% to 4.2%, and 9.4% to 16.1% in early (T1) PDC [7,15,23]. The findings from several studies have shown the rate of LNM to be as low as 0% under certain conditions, and have proposed ESD as an alternative to surgical gastrectomy. Li et al. [11] and Kunisaki et al. [15] similarly proposed endoscopic resection in patients with PDC limited to mucosa, less than 20 mm in diameter and without LVI, on the basis that no LNM was detected in such a cohort. Park et al. [23] also reported that patients with PDC that was less than 15 mm in size, confined to the mucosa, or showing minimal submucosal infiltration (≤500 μm) had no LNM and therefore should be considered for ESD. Lee et al. [7] reported that independent risk factors for LNM in poorly differentiated EGC were submucosal invasion, a tumor size greater than 2 cm, presence of LVI, and female sex; therefore, ESD should be carefully considered in such patients. In contrast, other studies showed that PDC possesses clinicopathological features with a higher risk of LNM [13], and had a lower en-bloc resection and complete resection rate compared to signet ring cell EGC [13,29] and supported that PDC should not be considered for endoscopic resection.

Previous studies have generally compared early cases of PDC, including those that have invaded the submucosa, based on pathological information from surgically resected tissues obtained from relatively small numbers of patients, and they tended to focus on the risk factors associated with LNM in early PDC. However, previous studies have commonly proposed submucosal invasion as a significant risk factor predicting LNM [7,15,23,24], and another study reported that PDC had a higher rate-positive vertical margin and submucosal invasion than positive lateral margin and mucosal confinement, compared with signet ring cell EGC [27]. Based on these findings, mucosa-confined PDC have a lower LNM rate, but there has been no detailed analysis of these patients to date. Therefore, we attempted to identify the clinicopathological factors predictive of LNM in intramucosal PDC and furthermore, developed a systematic approach toward aggregating the different risk factors. To the best of our knowledge, the present study is the first study to identify the risk factors in intramucosal PDC, including a relatively large number of patients. In the present study, multivariate analysis revealed that female sex, tumor size, invasion of depth, and LVI were significant factors for predicting LNM. This supports the result of the previous studies on PDC, which showed a significant correlation between the high incidence of LNM and tumor size, submucosal invasion, or the presence of LVI [7,15,23,24]. Furthermore, among the mucosa-confined PDC, we analyzed the depth of invasion subdivided in to two layers, namely the lamina propria and muscularis mucosa, in contrast to previous studies [7,13,15,23,24] that divided the depth of invasion into mucosa and submucosa. No study has determined the correlation between LNM and level of invasion within the mucosal layer, lamina propria, or muscularis mucosa, in EGC. Interestingly, in this study, we found a significant association between LNM and the relative invasion depth in the mucosal layer in PDC, a finding different from other types of EGC but similar to superficial esophageal cancer [30–32]. The cutoff value for the tumor size was somewhat larger compared to previous studies [7,11,15,23], in which majority of recent studies (73.3%) on ESD for undifferentiated EGC suggested that a diameter of 20 mm to 30 mm would be the upper limit of the criterion, based on their results of LNM [33]. However, the goal of our study was to develop a prediction model for LNM with a good discriminatory power, rather than finding the cutoff size without LNM. To guide treatment decision and select patients who may undergo ESD, the cutoff value for the tumor size that showed no LNM (1.0cm) should be used, for the oncologic safety. Regarding the female sex, although the nature of the link between sex and LNM remains unclear, this has been previously found to be predictive of LNM in early PDC [7], differentiated submucosal EGC [34], and depressed EGC [35]. According to this RSS model that incorporated the patient’s sex, tumor’s size, the tumor’s depth of invasion, and LVI to predict the LNM in intramucosal PDC, of the 130 patients with a total risk score of 0, only 1 (0.8%) had LNM, and of the 445 patients with a total risk score of 1, only 7 (1.6%) had LNM. Of the 594 patients with a total risk score of ≥2 (the high-risk group), 53 (8.9%) had LNM. Although our model had a relatively low PPV (8.9%), the NPV (98.6%) was very high, which will be particularly useful for identifying patients with low risks for LNM. Analyzing resected specimens who have undergone ESD, this RSS maybe applicable for making additional treatment after ESD.

This study had limitations. First, we only used conventional hematoxylin and eosin staining; therefore, it was difficult to precisely diagnose lymph-node micrometastasis. As lymph node micrometastasis might be one of the key causative factors for patients with recurrent gastric cancer [36,37], future studies that establish the lymph-node micrometastasis status, by using immunohistochemistry and reverse transcription-polymerase chain reaction, are essential for accurate diagnoses and reducing oncological risk [38]. Second, we did not perform an external validation, so concerns about generalizability are warranted. Once validated with an external data set, the prediction model may gain higher generalizability and may be useful for designing prospective studies.

In conclusion, because there is no definitive guideline to identify patients with intramucosal PDC at low risk of LNM, this RSS could be useful in clinical practice, and may be applicable for making final decision after ESD. To determine the recurrence, future prospective studies with long-term follow-up assessment are needed.

## Supporting Information

S1 Appendix Participant-level data. (XLSX)

S2 Appendix SAS code. (TXT)

S3 Appendix R code. (TXT)

S4 Appendix ROC curve of the prediction model fitted from the training set. (DOCX)

S1 Table Characteristics of the Training Set and Test Set. (DOCX)
