# Supplementary figures and images for: A Risk Prediction Model Based on Lymph-Node Metastasis in Poorly Differentiated–Type Intramucosal Gastric Cancer
Source: PLoS One. 2016 May 26;11(5):e0156207. doi: 10.1371/journal.pone.0156207 (PMC4881979; doi:10.1371/journal.pone.0156207)

**Supplementary Information 5.** ROC curve of the prediction model fitted from the training set


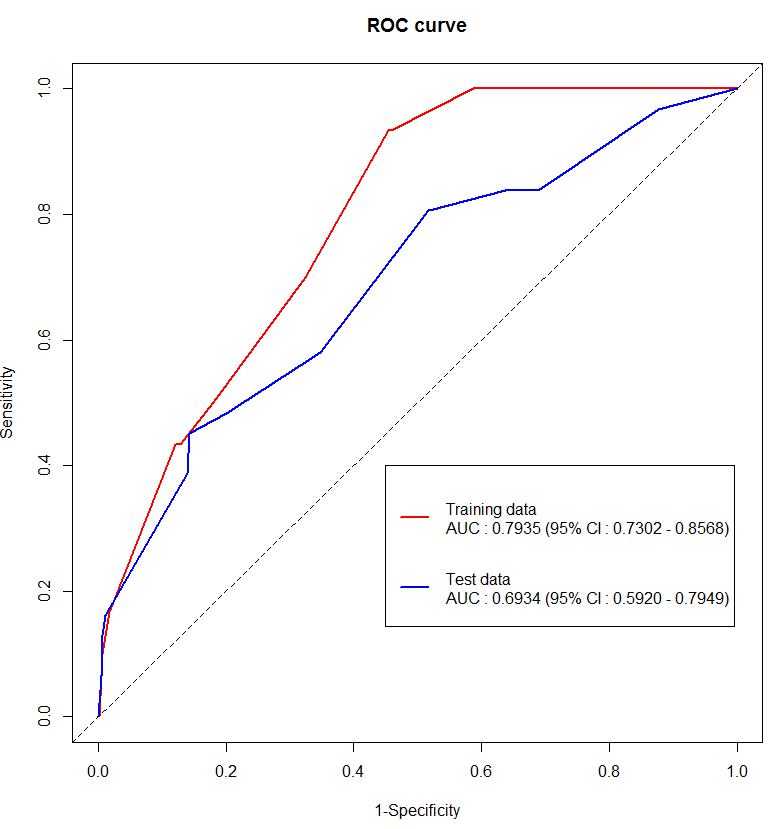

Supplement: S4 Appendix — (DOCX) [file pone.0156207.s004.docx]
