# Supplementary material for: A Risk Prediction Model Based on Lymph-Node Metastasis in Poorly Differentiated–Type Intramucosal Gastric Cancer
Source: PLoS One. 2016 May 26;11(5):e0156207. doi: 10.1371/journal.pone.0156207 (PMC4881979; doi:10.1371/journal.pone.0156207)
Supplement: S1 Table — (DOCX) [file pone.0156207.s005.docx]

**Supporting Table 1.** **Characteristics of the Training Set and Test Set**

|  | **Training set**  **(N = 584)**  **% (n)** | **Test set**  **(N = 585)**  **% (n)** | ***P*-value*** |
| --- | --- | --- | --- |
| Age (mean±SD) | 52.6 ± 10.7 | 52.9 ± 11.1 | 0.874 |
| Sex (male:female) | 322 : 262 | 312 : 273 | 0.536 |
| Tumor location  Upper  Middle  Lower | 9.4 (55)  43.7 (255)  46.9 (274) | 9.1 (53)  45.0 (263)  46.0 (269) | 0.902 |
| Macroscopic type  Elevated  Flat  Depressed  Mixed | 1.5 (9)  16.6 (97)  54.1 (316)  27.7 (162) | 2.6 (15)  21.2 (124)  51.6 (302)  24.6 (144) | 0.104 |
| Ulcer  No  Yes  Tumor size | 93.8 (548)  6.2 (36)  2.9 ± 2.1 | 95.0 (556)  5.0 (29)  3.1 ± 2.0 | 0.368  0.036 |
| Depth of invasion  Lamina propria  Muscularis mucosa | 33.2 (194)  66.8 (390) | 34.4 (201)  65.6 (384) | 0.680 |
| Number of resected nodes(mean±SD) | 39.7 ± 13.3 | 41.2 ± 14.1 | 0.229 |
| LVI  No  Yes | 96.4 (563)  3.6 (21) | 97.3 (569)  2.7 (16) | 0.401 |

*By logistic regression analysis.

LNM indicates lymph node metastasis; SD, standard deviation; LVI, lymphatic-vascular involvement.
